# Tackling the outbreak of nipah virus in Bangladesh amidst COVID‐19: A potential threat to public health and actionable measures

**DOI:** 10.1002/hsr2.2010

**Published:** 2024-03-27

**Authors:** Nadim Sharif, Nazmul Sharif, Afsana Khan, Shuvra K. Dey

**Affiliations:** ^1^ Department of Microbiology Jahangirnagar University Savar Bangladesh; ^2^ Department of Mathematics Rajshahi University of Engineering & Technology Rajshahi Bangladesh; ^3^ Department of Statistics Jahangirnagar University Savar Bangladesh

**Keywords:** annual outbreaks, Bangladesh, fruit bat, nipah virus, risk factors

Nipah virus of family paramyxovirus is a highly virulent infectious pathogen with epidemic potential.^1^ Nipah virus is a zoonotic (pig, cattle), mainly a bat‐borne (*Pteropus*)^1^, ^2^ pathogen reported in South to Southeast Asia^1^, ^2^, ^3^, ^4^ that can be transmitted between humans. Bat‐to‐human transmission of the virus is reported every year only from Bangladesh.^1^, ^2^, ^3^, ^4^, ^5^ With no effective treatment or vaccines and a case fatality rate of about 75%, the World Health Organization (WHO) has defined nipah virus as priority pathogen with a high risk of epidemic potential.^4^, ^5^ Sudden seasonal sporadic cases and smaller outbreaks have been reported continuously in various localities in Bangladesh since 2001.^3^, ^4^, ^5^ The effective intervention to contain larger outbreaks requires an understanding of the mechanism of breaching of virus host barrier and transmission to human.

Nipah virus was first identified in Malaysia during 1998−1999.^5^, ^6^ Since the first report, about 700 cases have been reported worldwide.^3^, ^4^, ^5^, ^6^ Due to the abundance of known reservoir of nipah virus, the countries in Southeast Asia including India, Bangladesh, Myanmar, Cambodia, Ghana, Indonesia, Madagascar, the Philippines, and Thailand are at high risk of outbreaks.^5^, ^6^ From 2001 to 2023, a total of 333 cases (47% of the global cases) and 237 fatalities with a case fatality rate of 71.2% have been reported in Bangladesh (Figure 1).^5^, ^6^, ^7^ The highest number of cases (67) were reported in 2004, followed by 43 in 2011, 37 in 2014, 31 in 2013, 18 in 2007 and 2010, 17 in 2012, and 15 in 2015, respectively (Figure 1).^7^, ^8^ In recent 3 years, seven cases in 2020 and two in 2021 and 11 in 2023 have been reported in Bangladesh.^6^, ^7^, ^8^ The largest number of fatality (50) was also documented in 2004, followed by 37 in 2011, 25 in 2013, 16 in 2010 and 2014, respectively (Figure 1).^5^, ^6^, ^7^, ^8^ The actual burden of nipah virus is significantly higher than the reported cases and fatalities in Bangladesh due to lack of real‐time and sensitive countrywide surveillance.

**Figure 1:**
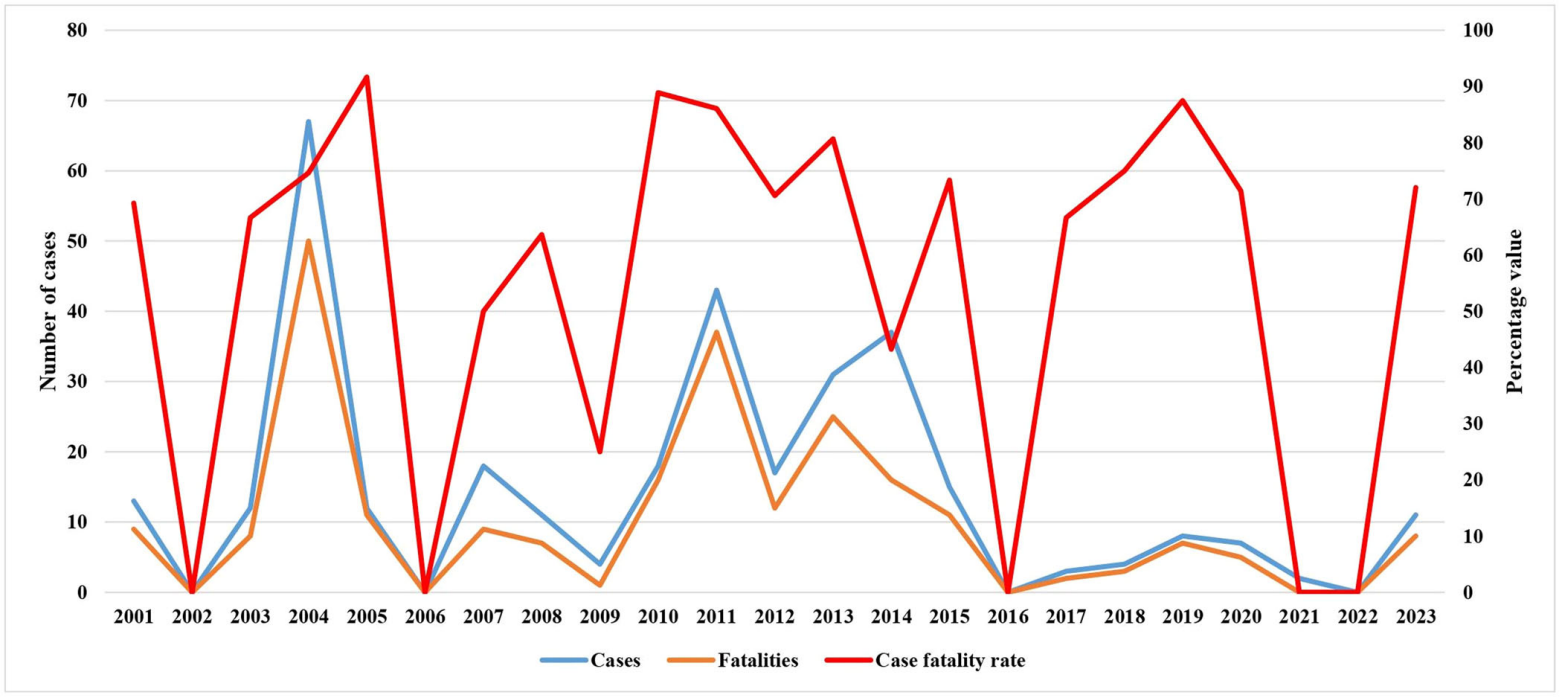
Trends of confirmed cases, fatalities, and case fatality rate of nipah virus in Bangladesh during 2001−2023.

The first case was documented from Meherpur,^4^ the northwestern district in Bangladesh during 2001. Out of 64 districts, sporadic cases have been found in 32 districts (50%).^5^, ^8^ The highest number of cases (67 and 43 confirmed cases) and fatalities (50 and 37) was reported from Faridpur, a central district in 2004 and 2011, respectively (Supporting Information S1: Figure I).^7^, ^8^ About 21−30 confirmed cases from Lalmonirhat, Naogaon, (the northern districts), and Rajbari were reported individually. Confirmed cases were found mostly from the central and northern districts in Bangladesh.^7^, ^8^ The first identified probable risk factor was close contact with sick cow in Meherpur in 2001 followed by probable person‐to‐person transmission.^1^, ^4^, ^7^ After that, close contact with pig herds, climbing trees contaminated with feces of bats followed by human‐to‐human transmission were detected as source of infection in 2003 in Naogaon and 2004 in Rajbari, respectively. The largest outbreak in Faridpur in 2004 was started from probably drinking raw date sap and contaminated droplets or fomites from infected persons.^1^, ^4^, ^7^, ^8^ Drinking raw date palm sap contaminated with bat droppings or saliva was first identified as probable source of nipah virus in 2005 in Tangail.^4^ Among 333 cases, 223 (68%) were infected from spillover, animals, or unidentified source and 110 (32%) were infected from person‐to‐person.^1^, ^4^, ^7^, ^8^ However, both the sources of unreported and reported cases require more specific investigation to accurately identify the risk factors.

The limited epidemiological survey documented that nipah virus infection are most prevalent in male (~65%)^1^ and about 40% of the cases are reported among patients aged ≤18 years in Bangladesh.^1^, ^7^ About 90% case patients required hospitalization and 60% died before 7 days from the onset of the symptoms.^1^ Among the clinical manifestations, fever (100%) is the most common followed by altered mental status 85%, sever weakness (72%), difficulty in breathing (64%), vomiting (55%), and cough (52%), respectively.^1^, ^3^ The high case fatality rate, continuous outbreaks, and seasonal sporadic cases and the lack of adequate knowledge of the sources of the transmission requires urgent attention to avoid future health crisis.

The first case of COVID‐19 pandemic was documented on March 8, 2020 in Bangladesh.^9^ About two million cases and 30,000 deaths are reported from Bangladesh during 2020 and 2023.^9^ During the pandemic like most other countries, the health sector in Bangladesh was significantly affected. Majority of the efforts, strategies, and dedication were given for the prevention and management of COVID‐19 cases.^10^ Thus, the pandemic has contributed to a significant lack in the healthcare facilities.^10^ As a result, cases of nipah virus, WHO defined priority pathogen, received lack of priority and underreporting due to diversion of health facilities towards COVID‐19. Further, the significant proportion of overlapping symptoms of nipah virus and COVID‐19 infection has made it difficult to conduct differential diagnostic during the pandemic. The emergency situation during the COVID‐19 pandemic also made it difficult to identify any coinfection by both nipah and COVID‐19.

Climate changes may also have direct impact on the seasonal outbreaks of nipah virus in Bangladesh. One of the well identified sources of transmission of nipah virus is contaminated date sap, that becomes available in winter (December to February) in Bangladesh. As a traditional drink, many people in rural areas take raw date sap contaminated with bat spill‐over or saliva and get infected by nipah virus (Supporting Information S1: Figure II).

The national surveillance of nipah virus is not strong and available in only limited number of hospitals covering small regions in Bangladesh. Furthermore, the health system is not sufficiently designed to conduct operational surveillance across the country. Moreover, genomic surveillance data are insufficient to understand the evolutionary dynamics in Bangladesh. Globally, there is no approved treatment or vaccine against nipah virus. Preventive measures include policy of reduced exposure to infected bat, animal, and human to minimize transmission. Some local preventive measures such as using protection of cloth net in the clay pot during overnight date sap collection may reduce contamination by bat saliva or spill‐over. Further, programs to build public awareness to avoid drinking raw date sap must be undertaken regularly countrywide.

We have identified three major gaps related with public health concern of nipah virus outbreak in Bangladesh. First, cases of nipah virus with 72% case fatality rate is occurring every year in Bangladesh; second, lack of a strong countrywide surveillance and management system dedicated to nipah virus outbreak and insufficient genetic characterization; third, lack of regular campaign on building awareness about preventive measures among general people, specifically training of raw date sap collectors to use protection during overnight collection and lack of emergency response health facilities to tackle a larger outbreak and reduce health burden. In conclusion, the authorities should take integrated public health measures and emphasize on scatteredly reported nipah virus with utmost importance before it becomes a major health burden.

## AUTHOR CONTRIBUTIONS

**Nadim Sharif**: Conceptualization; investigation; writing—original draft; methodology; writing—review and editing; formal analysis; supervision; data curation; project administration; validation; software. **Nazmul Sharif**: Investigation; writing—review and editing; methodology; software; formal analysis. **Afsana Khan**: Conceptualization; methodology; validation; visualization; software; formal analysis; data curation. **Shuvra K. Dey**: Supervision; writing—review and editing; visualization; validation; project administration; resources; software; formal analysis. All authors have read and approved the final version of the manuscript.

## CONFLICT OF INTEREST STATEMENT

The authors declare no conflict of interest.

## TRANSPARENCY STATEMENT

The lead author Nadim Sharif and Shuvra K. Dey affirms that this manuscript is an honest, accurate, and transparent account of the study being reported; that no important aspects of the study have been omitted; and that any discrepancies from the study as planned (and, if relevant, registered) have been explained.

## Supporting information

Supporting information.

## Data Availability

The authors confirm that the data supporting the findings of this study are available within the article and its supplementary materials. Nadim Sharif & Shuvra K. Dey had full access to all of the data in this study and take complete responsibility for the integrity of the data and the accuracy of the data analysis.

## References

[1] Nikolay B , Salje H , Hossain MJ , et al. Transmission of Nipah virus—14 years of investigations in Bangladesh. N Engl J Med. 2019;380(19):1804‐1814. 31067370 10.1056/NEJMoa1805376 PMC6547369

[2] Whitmer SLM , Lo MK , Sazzad HMS , et al. Inference of Nipah virus evolution, 1999–2015. Virus Evol. 2021;7(1):veaa062. 34422315 10.1093/ve/veaa062 PMC7947586

[3] Luby SP . The pandemic potential of Nipah virus. Antiviral Res. 2013;100(1):38‐43. 23911335 10.1016/j.antiviral.2013.07.011

[4] Rahman M , Chakraborty A . Nipah virus outbreaks in Bangladesh: a deadly infectious disease. WHO South‐East Asia J Public Health. 2012;1(2):208. 28612796 10.4103/2224-3151.206933

[5] WHO . Nipah virus fact sheet. Accessed October 14, 2022. https://www.who.int/news-room/fact-sheets/detail/nipah-virus

[6] CDC . Nipah virus. Accessed October 14, 2022. https://www.cdc.gov/vhf/nipah/index.html

[7] Institute of Epidemiology Disease Control and Research . Nipah virus transmission in Bangladesh. 2023. Accessed October 10, 2023. https://iedcr.gov.bd/surveillances/93c87e70-9c22-4f21-9506-a1161ecf404f

[8] The Business Standard. As winter nears, many in Bangladesh fear a Nipah virus re‐emergence. 2021. Accessed October 14, 2022. https://www.tbsnews.net/bangladesh/health/winter-nears-many-bangladesh-fear-nipah-virus-re-emergence-334861

[9] Coronavirus (COVID‐19) update—DGHS . 2023. Accessed October 10, 2023. https://dghs.gov.bd/index.php/en/home/5343-covid-19-update

[10] Sharif N , Alzahrani KJ , Ahmed SN , et al. Protective measures are associated with the reduction of transmission of COVID‐19 in Bangladesh: a nationwide cross‐sectional study. PLoS One. 2021;16(11):e0260287. 34807962 10.1371/journal.pone.0260287 PMC8608304

