# Supplementary material for: Tackling the outbreak of nipah virus in Bangladesh amidst COVID‐19: A potential threat to public health and actionable measures
Source: Health Sci Rep. 2024 Mar 27;7(4):e2010. doi: 10.1002/hsr2.2010 (PMC10973550; doi:10.1002/hsr2.2010)
Supplement: Supplementary file 1 — Supporting information. [file HSR2-7-e2010-s001.docx]

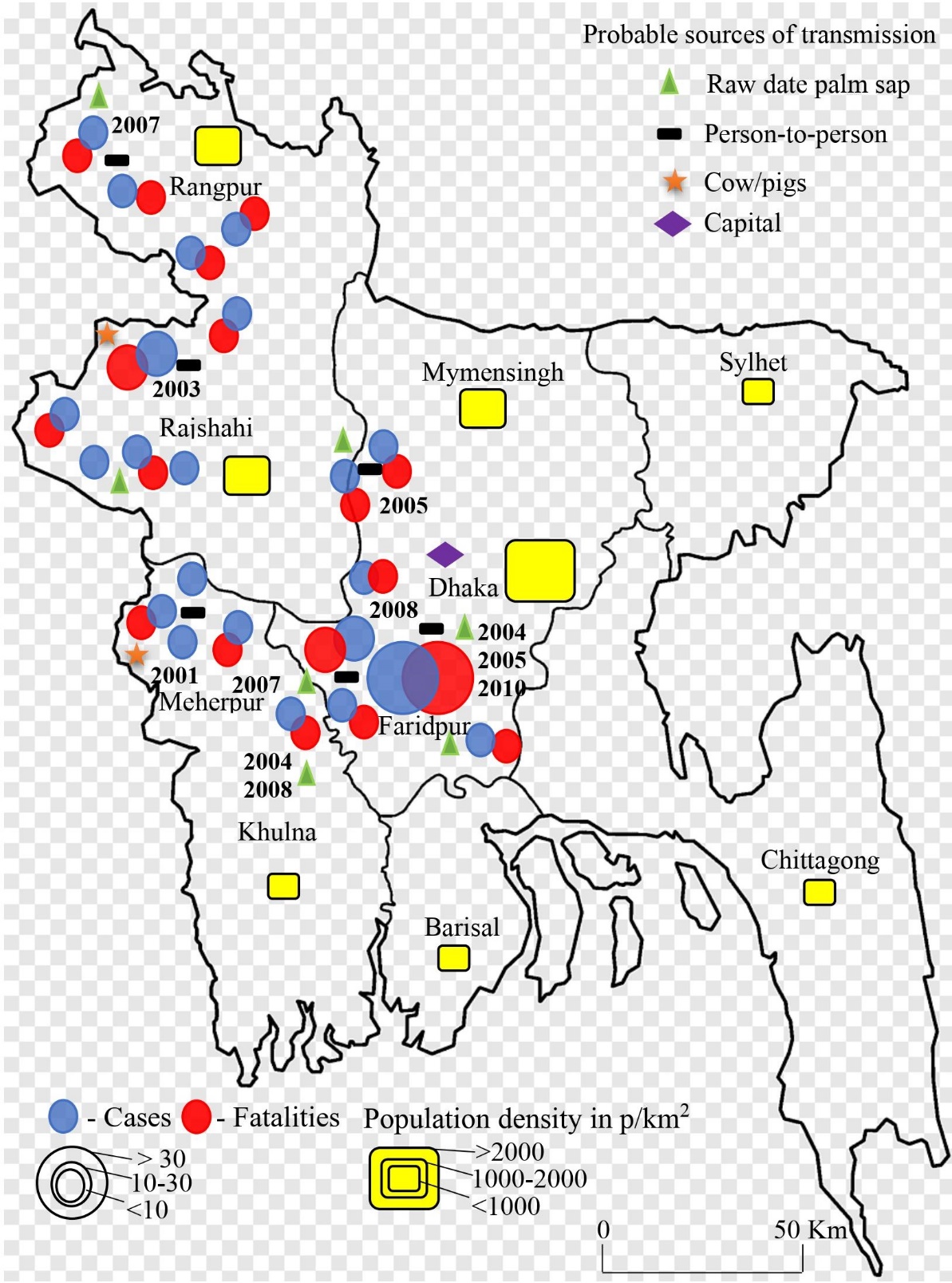


**Supplementary Figure IA.** Countrywide distribution of confirmed nipah virus cases, fatalities with probable source of infection in Bangladesh during 2001-2010.


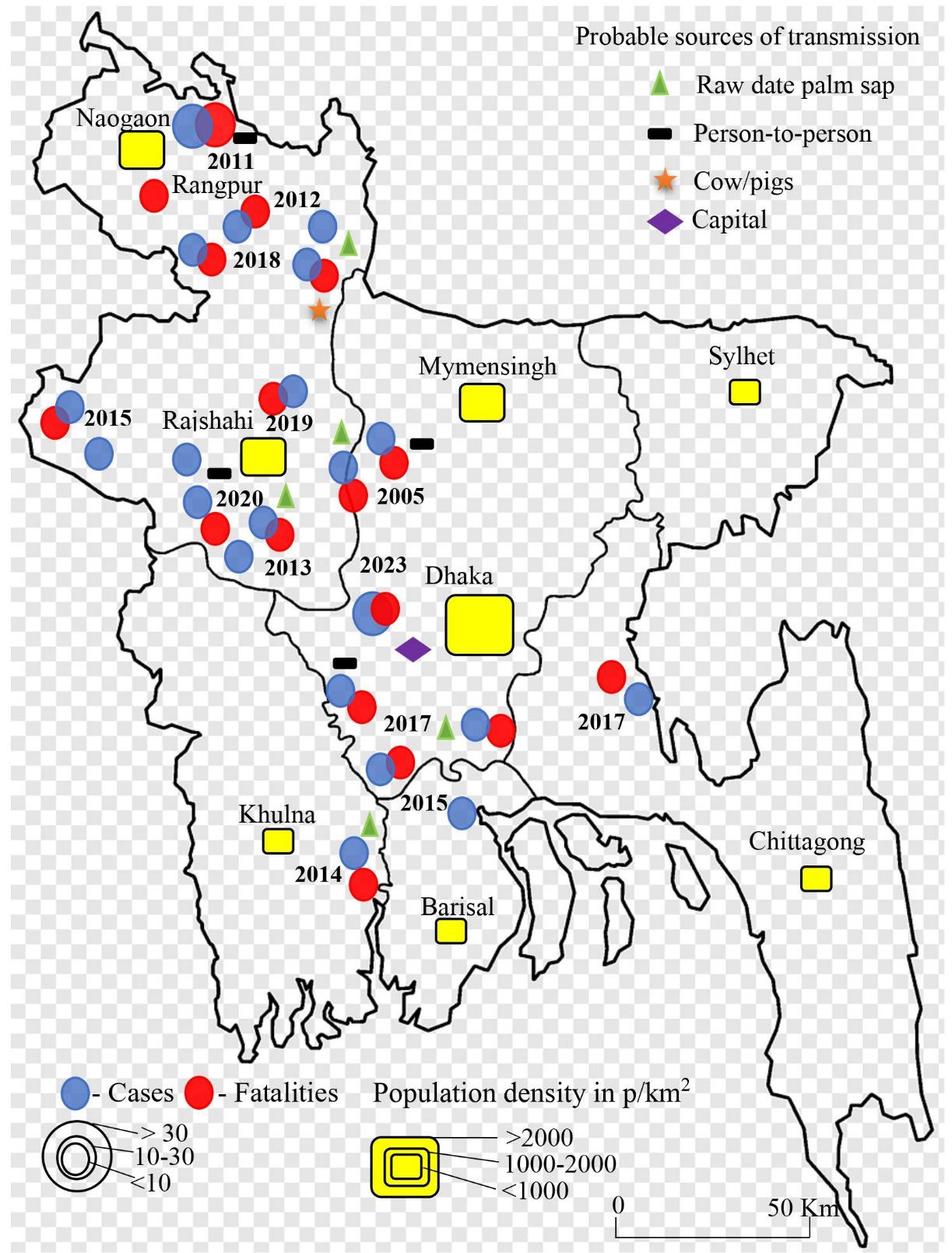


**Supplementary Figure IB**. Countrywide distribution of confirmed nipah virus cases, fatalities with probable source of infection in Bangladesh during 2011-2023. Person-to-person transmission included direct contact with infected persons, saliva, nasal and oral droplets, and dead body.

**
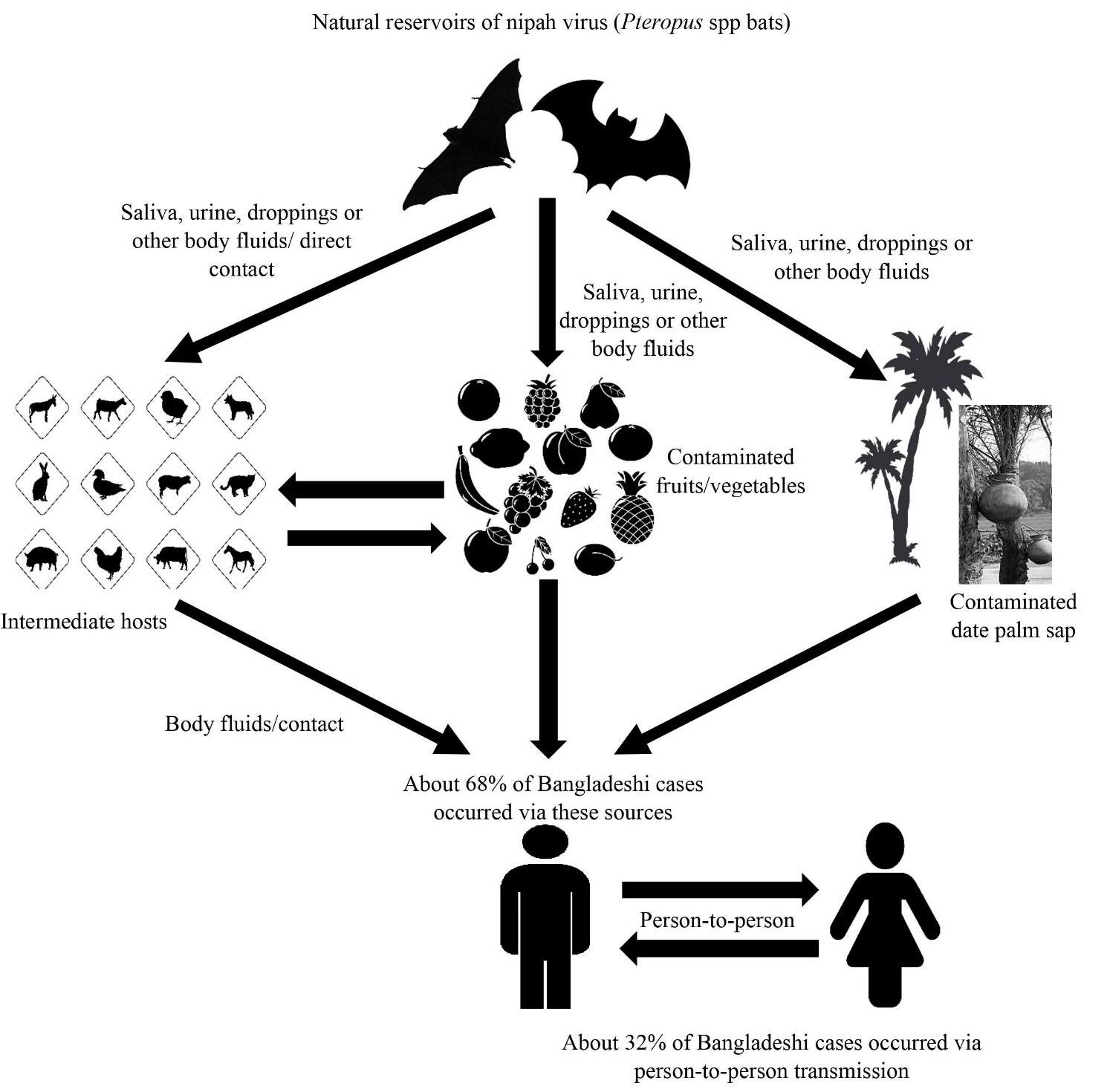
**

**Supplementary Figure II**. Origin and probable sources of transmission of nipah virus in Bangladesh
